# Supplementary material for: New Insights and Methods for Recording and Imaging Spontaneous Spreading Depolarizations and Seizure-Like Events in Mouse Hippocampal Slices
Source: Front Cell Neurosci. 2021 Nov 26;15:761423. doi: 10.3389/fncel.2021.761423 (PMC8663723; doi:10.3389/fncel.2021.761423)
Supplement: Supplementary file 2 [file Data_Sheet_1.PDF]

Supplementary Material

1 Supplementary Figures

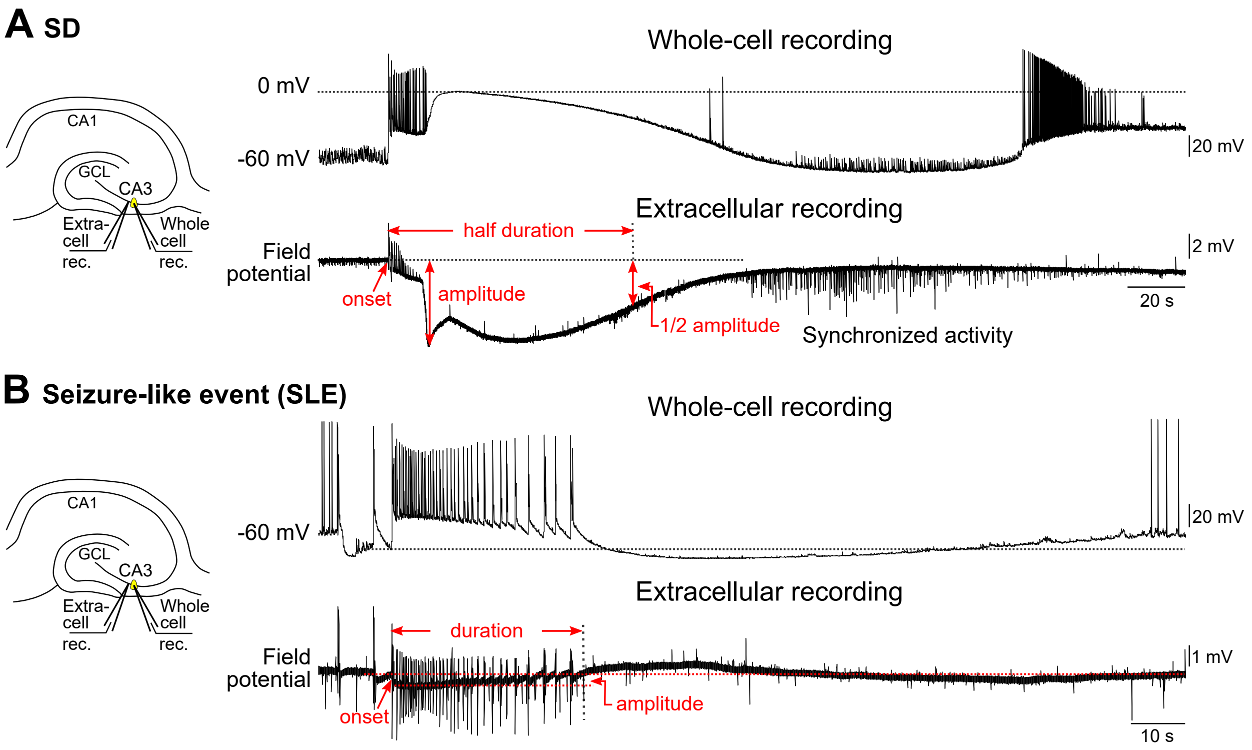

Supplementary Figure 1. Schematic illustrates the quantification for SD (A) and SLEs (B). All measurements used field potential recordings. **(A)** The onset of an SD was marked by beginning of a train of bursts that was followed by a sudden, large depolarization in whole cell recording or a large negative deflection in the field recording. The amplitude of an SD was measured as the maximal negative deflection relative to baseline. The half duration of SD was from the onset of the SD to the point where the recovery of SD reached half of its maximal amplitude. **(B)** The onset of an SLE was the beginning of the sudden large depolarization in whole cell recording, which corresponded to the initial burst in the field recording. The duration was the difference between the onset and the point where the SLE returned to baseline. The amplitude was the maximal negative deflection during the high-frequency bursts, which corresponded to the sustained firing in the whole cell recording.

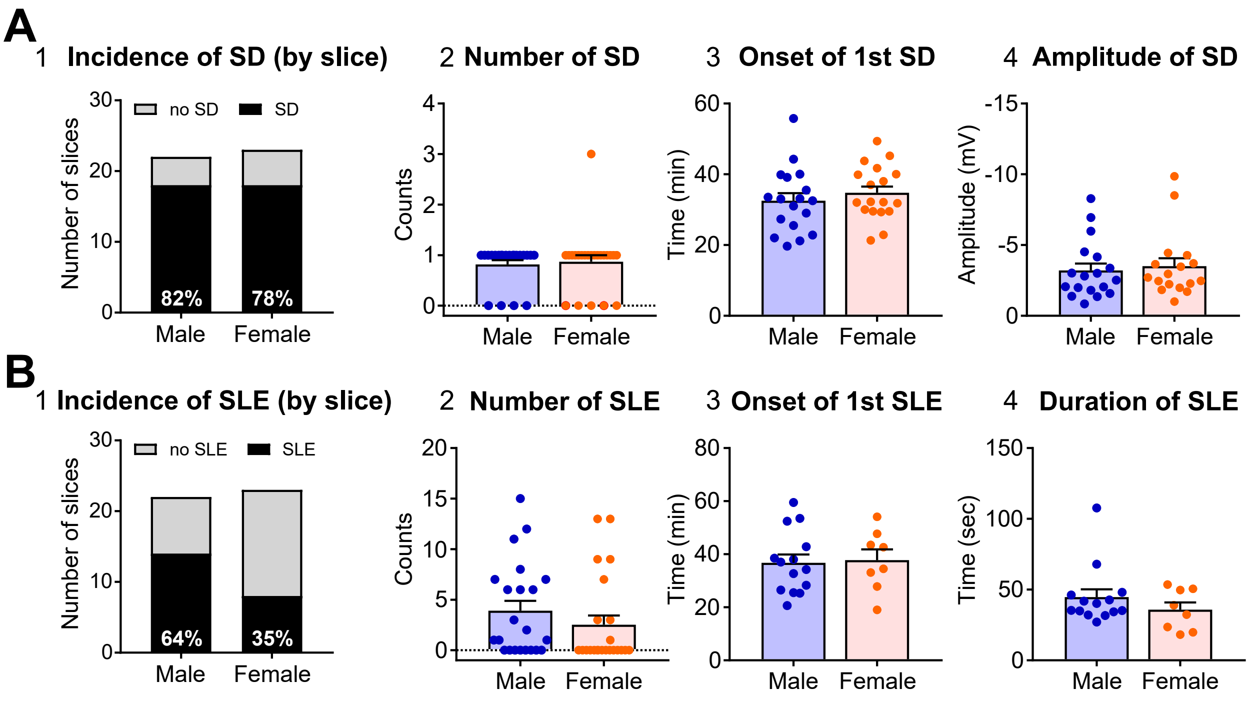

Supplementary Figure 2. Lack of sex difference in characteristics of SD and SLEs in the first 60-min of 0 Mg^{2+}/5 K^{+} aCSF exposure. **(A)** Quantification of SD. No statistical difference was found between males and females in the incidence of SD (A1, male, 22 slices/12 mice; female, 23 slices/10 mice; Fisher's exact test, $p > 0.9$), number of SDs (A2, male, 22 slices/12 mice; female, 23 slices/10 mice; Mann-Whitney test, $U = 253$, $p > 0.9$), onset of the 1st SD (A3, male, 18 slices/10 mice; female, 18 slices/9 mice; Unpaired t test, $t(34) = 0.80$, $p = 0.43$), and amplitude of SDs (A4, male, 18 slices/10 mice; female, 18 slices/9 mice; Mann Whitney test, $U = 137$, $p = 0.61$). **(B)** Quantification of SLEs. No statistical difference was found between males and females in the incidence (B1, male, 22 slices/12 mice; female, 23 slices/10 mice; Fisher's exact test, $p = 0.08$), the number (B2, male, 22 slices/12 mice; female, 23 slices/10 mice; Mann-Whitney test, $U = 191.5$, $p = 0.14$), the onset (B3, male, 14 slices/9 mice; female, 8 slices/5 mice; Unpaired t test, $t(20) = 0.20$, $p = 0.85$), and the duration of SLEs (B4, 14 slices/9 mice; female, 8 slices/5 mice, Mann-Whitney test, $U = 46$, $p = 0.53$).

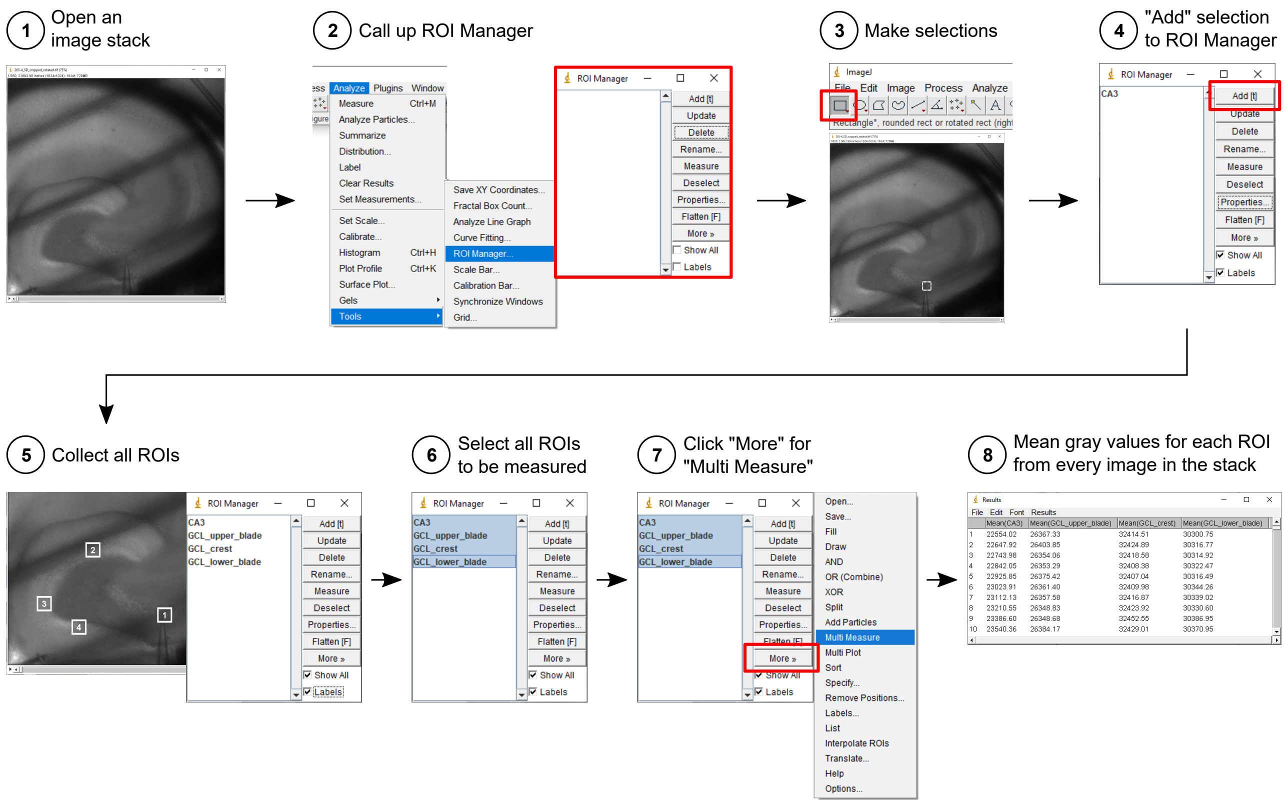

Supplementary Figure 3. Intrinsic optical signal analysis using ImageJ. **Step 1:** Images acquired throughout an SD were processed as an image stack. **Step 2:** Region of interest (ROI) manager was used to organize multiple ROIs. **Step 3:** ROIs were 50 pixels x 50 pixels squares, drawn by the rectangle tool. **Step 4:** Each ROI was added into ROI Manager. **Step 5:** CA3 (#1), upper blade of granule cell layer (GCL) (#2), crest of GCL (#3), and lower blade of GCL (#4) were analyzed. **Step 6:** Once all ROIs were collected, all ROIs were selected for analysis all at one time. **Step 7:** Analysis was made using the "Multi Measure" function. **Step 8:** Mean gray values of each ROI from every image in the stack were measured and displayed in the result window. The list of ROIs was saved and re-used with proper adjustment for anatomical locations for the next image stack

2 Supplementary Video

Supplementary Video. A recorded SD event is shown in a four-time faster fashion. Left: original intrinsic optical recording. Right: difference from baseline with pseudocolor. The timelapse images were recorded every 0.5 sec and replayed at the four-time of the speed. The propagation of SD wavefront is evident from CA3 to CA1 and to dentate gyrus.
